# Thrombotic Microangiopathy and Venous Thrombosis in a Patient With Anti-Neutrophil Cytoplasmic Antibody-Associated Vasculitis

**DOI:** 10.7759/cureus.11665

**Published:** 2020-11-23

**Authors:** Khanh Duong, Samantha Etienne, Roberto Collazo-Maldonado, Irina Lytvak

**Affiliations:** 1 Department of Internal Medicine, Methodist Dallas Medical Center, Dallas, USA; 2 Department of Nephrology, Methodist Dallas Medical Center, Dallas, USA; 3 Department of Pathology, Methodist Dallas Medical Center, Dallas, USA

**Keywords:** vasculitis, thrombotic microangiopathy, venous thrombosis, anti-neutrophil cytoplasmic antibody-associated vasculitis, anca

## Abstract

Antineutrophil cytoplasmic antibody (ANCA)-associated vasculitis (AAV) is a systemic pauci-immune small vessel vasculitis. Its various presentations make AAV diagnosis challenging. Here, we present a case of AAV with thrombotic microangiopathy (TMA) and deep venous thrombosis (DVT). An 82-year-old Hispanic woman presented to the emergency department with malaise, lower extremity pain, and oliguria for three days. Her vital signs were normal, and her physical examination was unremarkable. The initial laboratory revealed thrombocytopenia (18 x 10^3^/µL), elevated creatinine (8.35 mg/dL), high lactic acid dehydrogenase (1627.5 U/L), an international normalized ratio of 1.6, and an activated partial thromboplastin time of 49 seconds. Urinalysis showed microscopic hematuria and proteinuria, and peripheral smear revealed schistocytes. She was admitted with concern for TMA. Further workup revealed an antinuclear antibody titer of 1:80, an ADAMTS13 level of 62%, a rheumatoid factor level of 151.7 IU/L, and myeloperoxidase (MPO)-ANCA level of 173 AU/mL. A computed tomography scan of the chest/abdomen/pelvis revealed pulmonary fibrosis and multifocal consolidations. She was also found to have extensive DVT of the lower extremities. Renal biopsy revealed early changes of TMA with one cellular crescent. She was diagnosed with AAV based on the kidney and lung findings, as well as the high titer MPO-ANCA. Her platelet count and creatinine improved significantly following treatment with plasma exchange, steroids, and rituximab. Unfortunately, she was then found to have an acute bowel perforation and expired. Even though typically rare, an increased incidence of venous thromboembolism (VTE) and TMA has been reported in patients with AAV. Its prompt recognition and treatment by clinicians are critical to mitigate the unfavorable outcomes from this condition.

## Introduction

Antineutrophilic cytoplasmic antibody (ANCA)-associated vasculitis (AAV) is a systemic vasculitis with unknown etiology. AAV is a pauci-immune small vessel vasculitis and its various presentations can make diagnosis challenging. An increased incidence of venous thromboembolism (VTE) and thrombotic microangiopathy (TMA) associated with AAV have been reported. Here, we present a case of AAV with TMA and deep venous thrombosis (DVT).

## Case presentation

**Figure 1:**
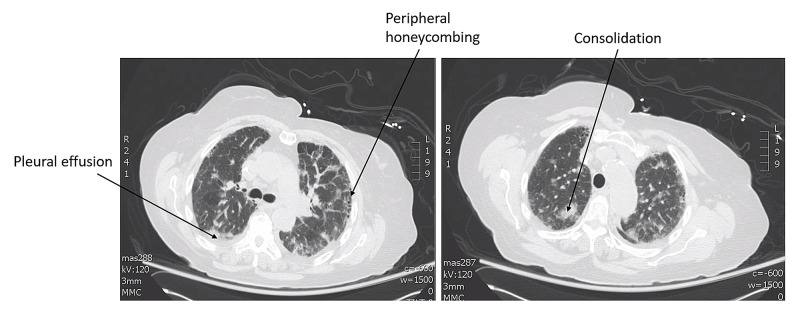
Computed tomography scan of the chest/abdomen/pelvis showed pulmonary fibrosis pattern, small bilateral perfusion, and multifocal areas of consolidation

An 82-year-old Hispanic woman with a past medical history of hypertension presented to the emergency department with malaise for three days. She also experienced nausea, vomiting, fatigue, lower extremity pain, shortness of breath, cough, and oliguria. No diarrhea, weight loss, history of malignancy, or kidney disease were reported. Her vital signs were normal, and her physical examination was unremarkable. The results of initial labs revealed low platelets with concomitant elevated creatinine, lactic acid dehydrogenase, international normalized ratio, and activated partial thromboplastin time; her haptoglobin level was undetectable (Table 1). A peripheral smear showed red blood cell schistocytes consistent with microangiopathic hemolytic anemia. Urinalysis showed microscopic hematuria and proteinuria. She was admitted with the diagnosis of microangiopathic hemolytic anemia and acute kidney injury. A non-tunneled dialysis catheter was placed and she underwent plasma exchange and was given steroids. Stool collection for *Escherichia coli* O157:H7 testing was not feasible as this patient did not have a bowel movement during hospital admission. She was later found to have an extensive lower extremity DVT, and heparin was administered. A workup for malignancy was ordered along with computed tomography (CT) scan of the chest/abdomen/pelvis. The CT scan revealed a pulmonary fibrosis pattern and multifocal areas of consolidation in the upper lung (Figure 1). Tumor markers (CEA, CA 19-9, CA 125, AFP) were negative. Rituximab was started as the patient’s ANCA results were positive. Her platelet count and creatinine levels improved significantly following treatment (Table 2).

**Table 1:** The patient’s initial workup ADAMTS13: a disintegrin and metalloproteinase with a thrombospondin type 1 motif, member 13; AFP: alpha-fetoprotein; ANA: antinuclear antibody; ANCA: antineutrophil cytoplasmic antibodies; aPTT: activated partial thromboplastin time; CA: cancer antigen; CEA: carcinoembryonic antigen; CRP: C-reactive protein; ENA: extractable nuclear antigen; ESR: erythrocyte sedimentation rate; GBM: glomerular basement membrane; HBsAg: hepatitis B surface antigen; Hb: hemoglobin; HCV: hepatitis C virus; HIV: human immunodeficiency virus; LDH: lactate dehydrogenase; MPO: myeloperoxidase antibodies; PT: prothrombin time.

Test name	Reference range	Lab value
Hematology		
Hb (g/dL)	12.0 - 16.0	14.5
Platelet count (×10^3^/µL)	130 - 400	18
Haptoglobin (mg/dL)	30 - 200	<20
LDH (U/L)	313 - 618	1627.5
D-Dimer, Quant (µg/mL)	0.00 - 0.50	>20.00
Fibrinogen (mg/dL)	214 - 481	92
aPTT (seconds)	23 - 37	49
PT	11.3 - 14.7	19.1
INR	0.9 - 1.2	1.6
Rheumatology panel		
ANA titer	Negative at 1:40	1:80
ENA antibodies screen (EU)	≤20.000	4.123
Anti-DNA antibody (IU)	≤25.000	14.220
Rheumatoid factor (IU/mL)	≤12.0	151.7
Cryoglobulin quant, blood	Negative	Negative
GBM Ab IgG (AU/mL)	0 - 19	0
Sm antibody (AU/mL)	0 - 40	5
Anti-Scl70 IgG (AU/mL)	0 - 40	0
Myositis antibody	Negative	Negative
ANCA IgG	<1:20	1:1280
MPO-ANCA (AU/mL)	0 - 19	173
PR3-ANCA (AU/mL)	0 - 19	1
Direct antiglobulin test		Negative
ADAMTS13	50-160%	62%
Inflammatory markers		
Procalcitonin (ng/mL)	<0.25	0.28
CRP (mg/L)	≤10	31
ESR (mm)	0 - 20	1
C3 (mg/dL)	88 - 165	121
C4 (mg/dL)	14 - 44	27
Infectious workup		
HIV Ab		Negative
HCV Ab		Negative
HbsAg		Negative
Blood culture		No growth at five days
Tumor markers		
CEA (ng/mL)	≤3.0	1.0
CA 19-9 (U/mL)	≤37.00	18.20
CA 125 (U/mL)	≤35	12
AFP (ng/mL)	<7.51	2.11
Others		
Vitamin B12 (pg/mL)	211 - 911	908
Folate (ng/mL)	3.0 - 20.0	17.6

**Table 2:** Kidney-function-and-platelet-level-monitoring-during-the-patient’s-length-of-stay

Test name	Reference range	Day 1	Day 2	Day 3	Day 4	Day 5	Day 6	Day 7
Creatinine (mg/dL)	0.7 - 1.3	8.35	6.92	4.90	3.46	2.96	2.20	1.80
Platelet (× 10^3^/µL)	130 - 400	18	9	42	108	127	112	226

**Figure 2:**
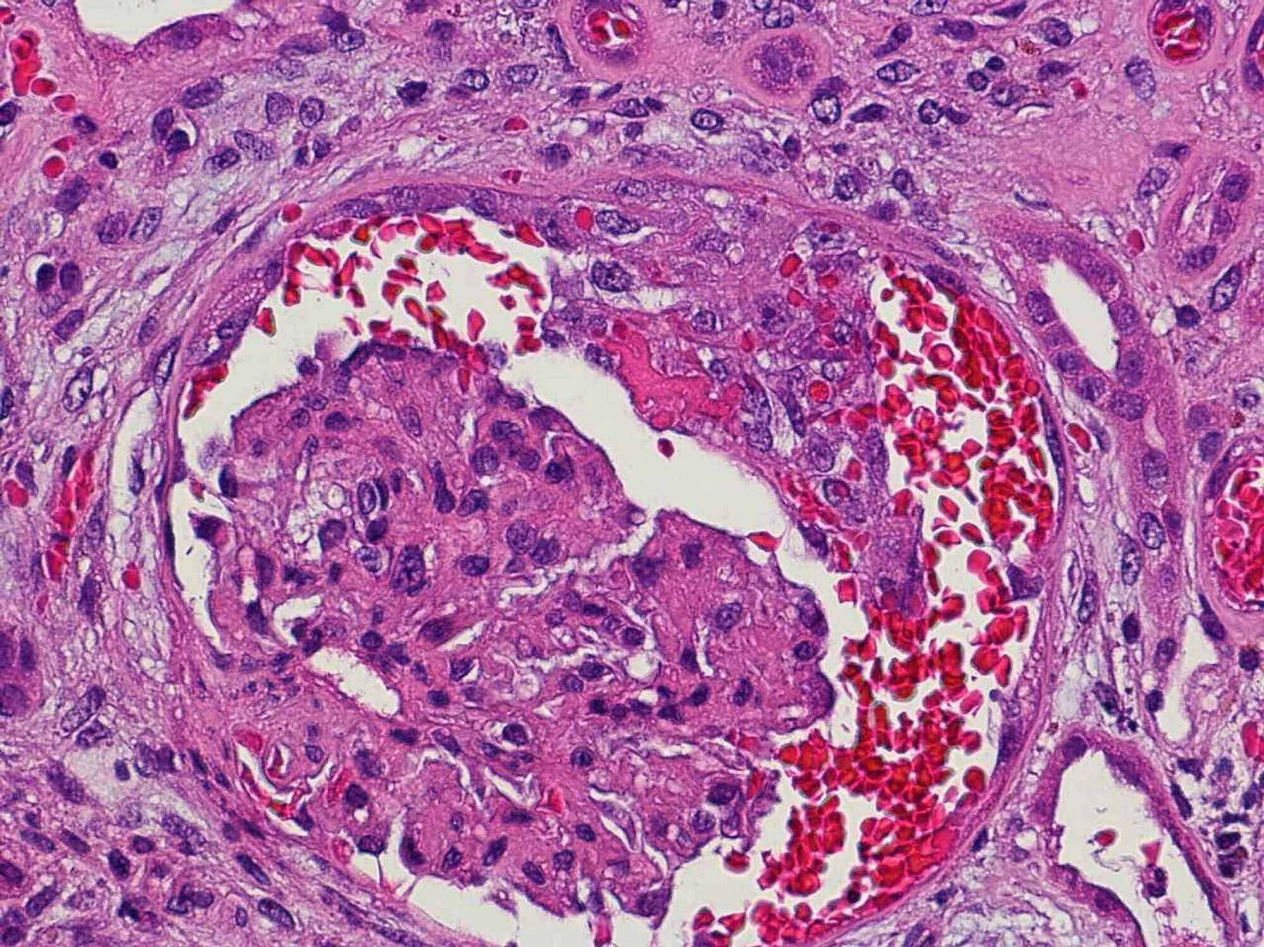
Kidney biopsy showed glomerulus with a cellular crescent (H&E stain)

A kidney biopsy was done following the resolution of her thrombocytopenia. Light microscopic examination of the kidney specimen revealed 23 glomeruli, of which six were globally sclerotic, one demonstrated early glomerular capillary thrombosis, and another demonstrated a cellular crescent (Figure 2). There was no evidence of vasculitis, but moderate arteriosclerosis was present. Mild interstitial fibrosis and tubular atrophy were found in the tubulointerstitium, involving 10% to 15% of the renal cortical tissue. Electron microscopy showed no electron-dense immune-type deposits or organized deposits. Immunofluorescent staining (IgG, IgA, IgM, C3, C1q, kappa, and lambda) of the glomeruli were negative.

During the course of treatment, this patient developed an acute bowel perforation not amenable to treatment and expired.

## Discussion

Our patient was admitted with TMA and DVT. At first, malignancy was an obvious option for the differential diagnoses; however, the workup was negative. An infection workup was also negative. This patient was diagnosed with AAV based on the kidney and lung findings as well as the very high titer of MPO-ANCA, consistent with microscopic polyangiitis (MPA). Pulmonary involvement is observed in 25% of patients with MPA, including diffuse alveolar hemorrhage, interstitial lung disease (ILD), and pleuritis [1,2]. ILD is present in about 7.2% of patients at the time of MPA diagnosis [1]. Approximately 80% of patients with MPA have renal manifestations [2]. Global glomerulosclerosis (defined as sclerotic changes in the glomerulus that compose >80% of the tuft) is common and can be seen in around 30% of MPA patients [3]. Glomerular crescents are present in approximately 65% of patients [3]. Findings in the kidney biopsy that support the AAV diagnosis in this patient included focal crescentic glomerulonephritis and global glomerulosclerosis using light microscopy and pauci-immune staining pattern using immunofluorescence. Vasculitis and fibrinoid necrosis are often seen in less than 20% of patients with MPA [3]; these were not seen in the biopsy specimen of this patient’s kidney.

TMA is characterized by abnormalities in the vessel wall of arterioles and capillaries that lead to microvascular thrombosis [4]. Primary TMA syndromes include thrombotic thrombocytopenic purpura, Shiga toxin-mediated hemolytic uremic syndrome (HUS), drug-mediated TMA, complement-mediated TMA (alternatively, atypical HUS), coagulation-mediated TMA, and metabolism-mediated TMA [5]. Secondary TMA can be seen in systemic infections, systemic cancer, severe preeclampsia, eclampsia, hemolysis, elevated liver enzymes, low platelet syndrome, severe hypertension, autoimmune disorders (e.g., systemic lupus erythematosus, systemic sclerosis, antiphospholipid syndrome), and hematopoietic stem-cell or organ transplantation [5]. Our patient had evidence of TMA on lab tests and kidney biopsy. Furthermore, lab tests/history revealed a normal ADAMTS13 level, normal vitamin B12 level, lack of diarrhea/infection, and no contributing medication use. The severe kidney involvement with evidence of TMA on the kidney biopsy made complement-mediated TMA a possible diagnosis. Her response to plasmapheresis and steroids also pointed to this diagnosis. Unfortunately, we failed to demonstrate complement dysregulation.

AAV-associated TMA has been described in several case reports [6-10]. The etiology is unknown, but the endovascular damage due to vasculitis and complement pathway abnormalities could be the triggers. AAV patients with concomitant renal TMA presented with more severe renal injury. Also, TMA was independently associated with all-cause mortality in patients with AAV [11]. Recent studies demonstrated an increased risk of thromboembolic events in AAV. The risk of VTE is over three times greater in patients with AAV than in the general population [12]. The risk of arterial thrombotic disease is also increased. The risk of cardiovascular disease is over three times greater and the risk for a cerebrovascular accident is eight times greater in patients with AAV than in the general population [12]. These risks are higher during the active phase, supporting the role of inflammation during the thrombosis in promoting a prothrombotic state [13].

Our patient responded very well to treatment; her creatinine and platelet levels normalized. Unfortunately, she developed acute bowel perforation. Bowel perforation has been reported to occur in about 15% of systemic small and medium-sized vessel vasculitides with gastrointestinal tract involvement [14].

## Conclusions

Even though typically rare, an increased incidence of VTE and TMA has been reported in patients with AAV. To the best of our knowledge, this is the first report of a patient with AAV that developed both TMA and DVT concomitantly in the active phase. Prompt recognition and intensive treatment with steroids, plasmapheresis, and rituximab treatment are critical to mitigate unfavorable outcomes.
